# Electric and Magnetic Field-Assisted Orientational Transitions in the Ensembles of Domains in a Nematic Liquid Crystal on the Polymer Surface

**DOI:** 10.3390/ijms151017838

**Published:** 2014-10-02

**Authors:** Alexander M. Parshin, Vladimir A. Gunyakov, Victor Y. Zyryanov, Vasily F. Shabanov

**Affiliations:** 1 L. V. Kirensky Institute of Physics, Krasnoyarsk Scientific Centre, Siberian Branch of the Russian Academy of Sciences, Krasnoyarsk 660036, Russia; E-Mails: gun@iph.krasn.ru (V.A.G.); zyr@iph.krasn.ru (V.Y.Z.); dir@iph.krasn.ru (V.F.S.); 2 Department of Energy, Siberian Federal University, Krasnoyarsk 660041, Russia

**Keywords:** liquid crystal, polycarbonate, domain structure, electric field, magnetic field

## Abstract

Using electro- and magneto-optical techniques, we investigated orientational transitions in the ensembles of domains in a nematic liquid crystal on the polycarbonate film surface under the conditions of competing surface forces that favor radial and uniform planar alignment of nematic molecules. Having analyzed field dependences of the intensity of light passed through a sample, we established the threshold character of the orientational effects, plotted the calculated intensity *versus* magnetic coherence length, and compared the latter with the equilibrium length that characterizes the balance of forces on the polymer surface.

## 1. Introduction

Liquid crystal (LC) layers with the ordered structure are widely used in both fundamental research in the field of physics of condensed matter and fabrication of electronic devices [1]. Of special interest are the structures with the competing aligning effects of surfaces that bound an LC. The competing effects can be spatially separated, as at the local Freedericksz transition [2] in LC layers when the short-range forces induced by the surface tend to align the LC director parallel or perpendicular to it and the long-range dispersion forces of anchoring, orthogonally to this direction. As a result, the occurring LC orientation corresponds to the minimum free energy, which meets the boundary conditions specified at the surface. Varying the surface shaping conditions for LC aligning by, e.g., sputtering of SiO layers, one can obtain the competing effect of the short-range forces that favor LC alignment along needles or grooves in the evaporated glass plates [3]. The competing conditions can cause orientational bistability and memory effects in the plane-parallel nematic cells used in LC displays [4]. An electric field applied in the cell plane and linear flexoelectric anchoring make it possible to significantly broaden the ranges of LC layer switching parameters [5]. The competing effects of elastic, surface, and gravitational forces can lead to the formation of complex LC structures that represent domain ensembles on the bounding surfaces [6,7]. Among these structures are domain networks confined between two bounding surfaces, one forming a radial LC configuration and the other favoring homeotropic alignment. Under energy balance, a hybrid structure with the axially distorted director arises in the bulk of the LC layer [8]. The aforementioned structures promise to extend the class of LC objects; however, very special conditions for formation [9] and conservation of the alignment can make their application difficult.

The study of the *N*-(4-methoxybenzylidene)-4-butylaniline (MBBA) nematic oriented on profiled and smooth cleaned glass surfaces in a magnetic field showed that its behavior cannot be described within the theory of elasticity [10]. It was shown that the layer localized at the surface was nearly invariable upon rotation of one of the cell substrates and at applying a magnetic field to it. The authors attributed the alignment of the nematic to absorption of its molecules due to the anisotropic interaction between electric dipoles caused by LC polarizability and surface dipoles of the substrate. Later on, this interpretation was extended: the layer of adsorbed molecules was considered as a mobile system where the exchange by molecules between the bulk and the surface is implemented via absorption and desorption of LC molecules [11,12]. Nevertheless, despite the complex physicochemical processes occurring inside the layer of adsorbed molecules, orientation of the director in the cell bulk adiabatically follows the boundary conditions. The distorted LC film in a cell can therefore be described using a modified model based on the theory of elasticity, introducing the interface energy, and the ordering is adequately described by the expression for free energy with the boundary conditions. Of special interest are the LC films on polymer surfaces. For example, adsorbed nematic molecules on the polyvinyl alcohol or polyimide surface interact with the polymer, forming the easy orientation axis [13,14]. Superposition of the molecular interactions determines anchoring with the easy axis parallel to the mean orientation of adsorbed molecules. In other words, the adsorbed nematic molecules are adjusted to the polymer chains and the director of the LC bulk layer orients parallel to the director of the surface layer. In our recent study [15], nematic structures with domain ensembles on the polycarbonate (PC) films were observed and the radial surface configuration and planar alignment of the director in the bulk of the LC layer above domains were investigated by optical methods. It has been shown that in the analysis of orientational nematic structures on the PC surfaces it is necessary to consider the influence of adsorption effects.

Here, we report the results of electro- and magneto-optical investigations of the orientational transitions of nematics in the cells with domains on the PC surface and consider the competing aligning effects induced by the PC surface on the LC structure.

## 2. Results

**Figure 1:**
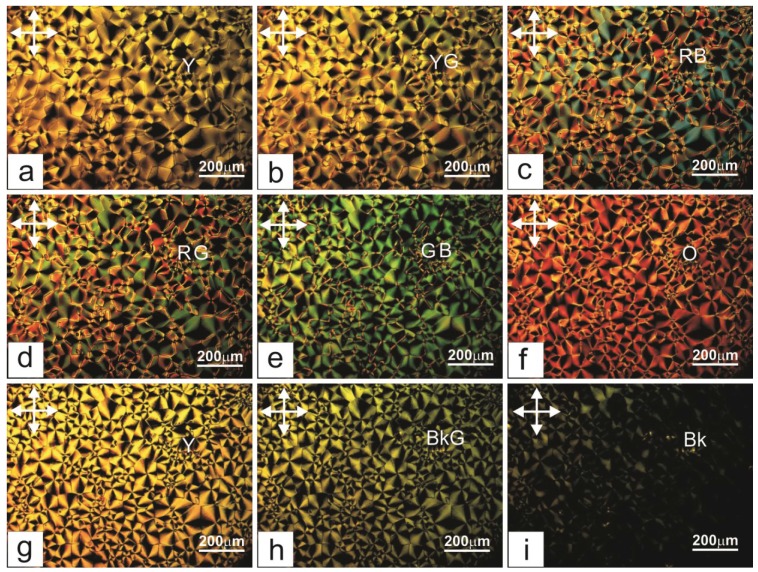
Microphotographs of domains in the 30 µm thick planar 5CB layer on the PC surface at applied voltages *U* of (**a**) 1.1; (**b**) 1.2; (**c**) 1.9; (**d**) 2.2; (**e**) 2.8; (**f**) 5; (**g**) 7; (**h**) 16, and (**i**) 30 V. Texture colors in an electric field are marked with characters Y(yellow), G (green), B (blue), R (red), О (orange), and Bk (black). Arrows show the polarization directions.

Figure 1 presents microphotographs of the texture of LC cells filled with the nematic LC 4-*N*-pentyl-4'-cyanobiphenyl (5CB) [15] aligned by the PC film in different electric fields applied perpendicularly to the layer. In the voltage range *U* ≤ 1.2 V, no variations were detected in the cell and the only observed texture was goldish-yellow with butterfly-shaped domains (Figure 1a). For *U* = 1.2 V, the texture color started intensively changing (Figure 1b) and various color combinations appeared (Figure 1c,h). The radial structures became pronounced. For certain *U* values, the visualized radial configurations were uniformly colored. In sufficiently strong electric fields, the radial domain network became green gradually and grew dark without changing its color (Figure 1h,i). For *U* > 30 V, the optically dark field image was established.

**Figure 2:**
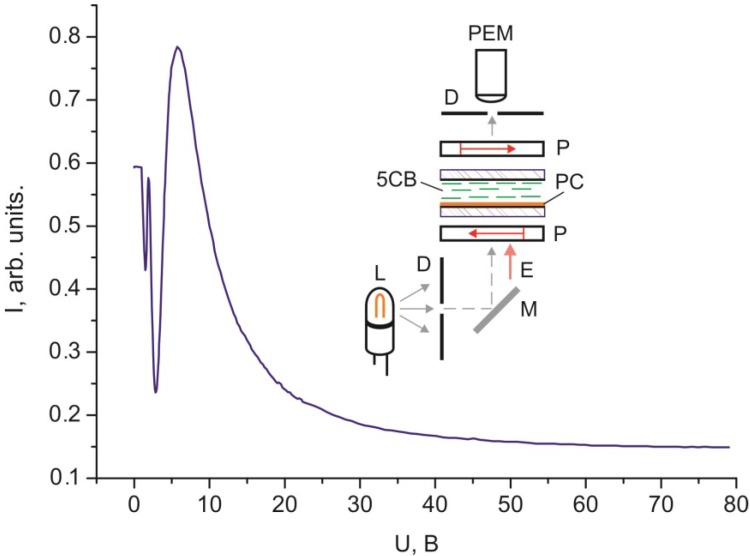
Dependence of intensity *I* of white light passed through the 30 µm thick planar 5CB layer with domains formed on the PC surface in crossed polarizers on voltage *U* of electric field *E*.

The above-described process is illustrated in Figure 2 by the dependence of light intensity *I* on electric voltage *U*. One can see the threshold *U* value, oscillations, and the monotonically descending portion at large voltages.

**Figure 3:**
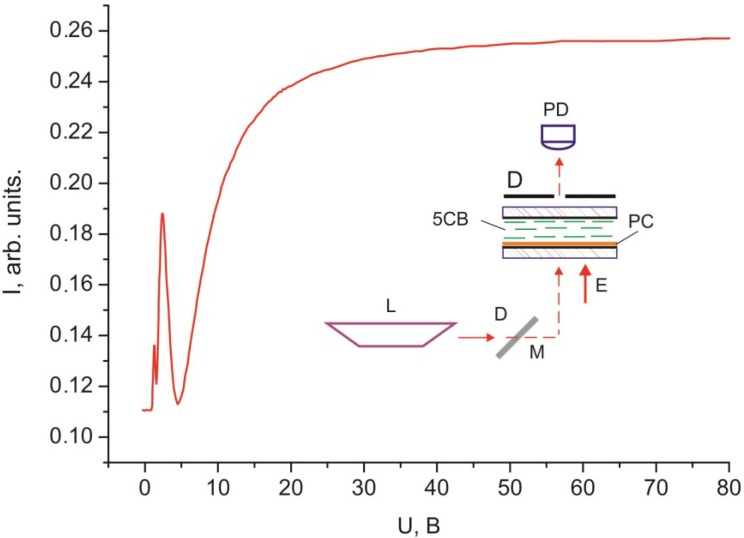
Voltage dependence of intensity *I* of monochromatic light passed through the 30 µm thick planar 5CB layer with domains formed on the PC surface.

**Figure 4:**
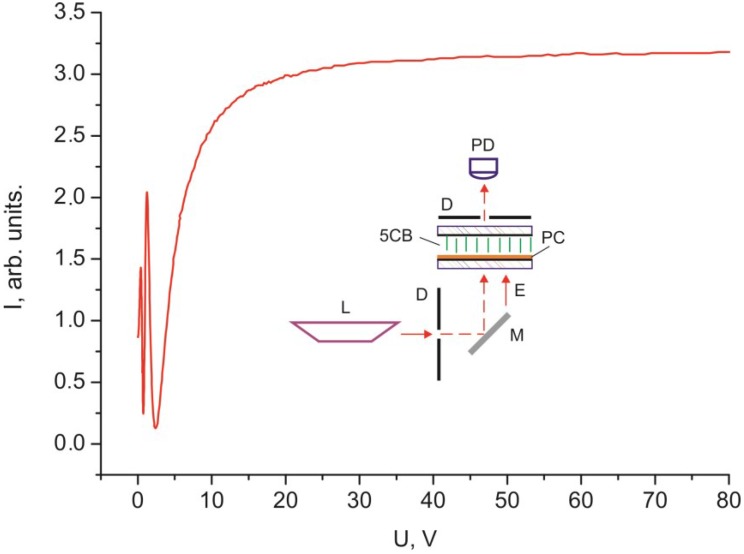
Voltage dependence of intensity *I* of monochromatic light passed through the 30 µm thick homeotropic 5CB layer with domains formed on the PC surface.

Figure 3 and Figure 4 show dependences of light intensity *I* for the 30 µm thick planar and homeotropic 5CB layers with domains formed on the PC surface that were scanned by a monochromatic laser radiation without polarizers. The dependences contain the threshold *U* value, the oscillating light intensity portion, and the monotonically ascending portion at large voltages.

**Figure 5:**
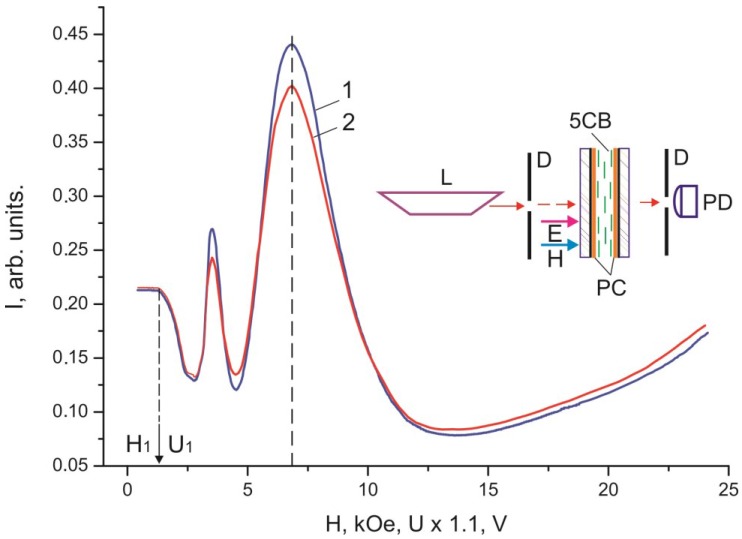
Dependences of intensity *I* of monochromatic light passed through the 30 µm thick planar 5CB layer formed in magnetic field H* on magnetic field *H* (curve 1) and voltage *U*(curve 2).

**Figure 6:**
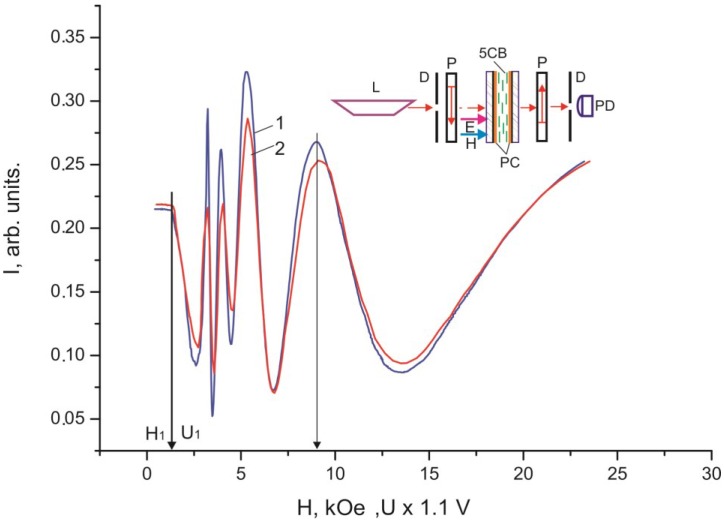
Dependences of intensity *I* of monochromatic light passed through the 30 µm thick planar 5CB layer formed in magnetic field H* on magnetic field *H* (curve 1) and voltage *U* (curve 2) in crossed polarizers.

Figure 5 and Figure 6 show dependences of light intensity *I* for the 30 µm thick 5CB layers confined between two PC films with domains on the surface, which were scanned by a monochromatic laser radiation, on electric field *E* and magnetic field *H* without polarizers and in crossed polarizers.

The applying voltage and magnetic field strength were scanned at 5 V/min and 5 kOe/min, respectively. At such scanning velocities, the dependences *I* (*U*, *H*) do not change under various cycles of field variations. This demonstrates the reversibility of the observed phenomenon.

## 3. Discussion

### 3.1. Director Field Distribution in the Liquid Crystal (LC) Layers on the Polycarbonate (PC) Surface

**Figure 7:**
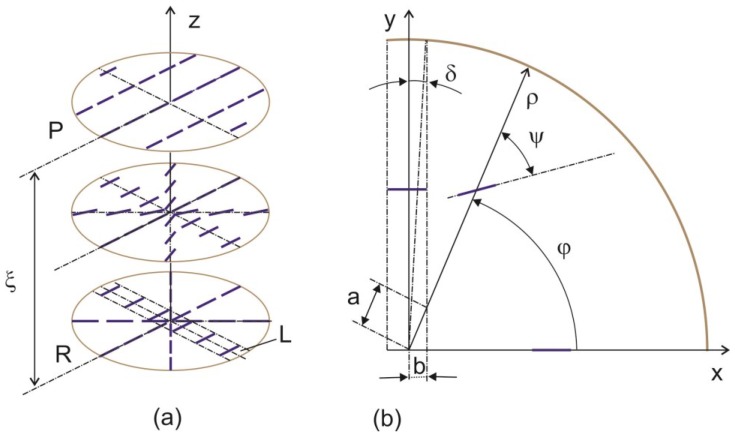
(**a**) Distribution of nematic director field *n* at the transformation of the radial structure to the uniform planar one at distance ξ from the polymer film surface and (**b**) parameters in the X0Y plane for calculation in cylindrical coordinates.

As was established in [15], the network of radial domains arises on the PC surface. The diameter of each domain is crossed by surface element *L* that tends to align perpendicularly to the director of the bulk nematic layer during the domain growth and can retain uniform planar alignment after the domain formation. We may assume that the structure forming in the investigated objects results from equilibrium between uniform alignment induced by *L* and the radial structure (Figure 7). At certain distance ξ from the surface, the radial structure gradually transforms to uniform alignment, since the latter should have the lower free energy due to the absence of strains. In the middle of Figure 7a, we present the LC molecule distribution in the transition layer of a domain.

We write the LC free energy of director *n* in volume *V* in the approximation of equal elastic constants *K* as [1].

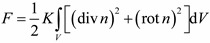
(1)

In the cylindrical coordinates, the nematic director components are written as

*n_ρ_* = −cosψ, *n_φ_* = cosψ, *n_z_* = 0 (2)

Angle ψ of deviation of molecules from polar radius ρ is zero on the surface (*z* = 0, ψ = 0) and ψ = φ at the distance *z* = ξ_0_ from the surface. We assume ψ = (1 − exp(*z*/ξ)·φ, taking into account the exponential weakening of the aligning effect of the surface at large distances [1]. Substituting director field distribution (2) in expression (1) and integrating over *l* ≤ ρ ≤ *R*, 0 ≤ φ ≤ 2π − 4δ, and 0 ≤ *z* ≤ ∞, we obtain

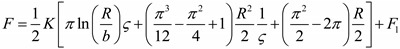
(3)

where *R* is the domain radius, *b* is the half-width of element L, *F*_l_ is the energy of distortion induced by L that contains the angular term calculated with regard to angle δ characterizing the angular size of L and the geometrical term related to the segment *a* = *b*/cos φ intercepted by element L from ρ.

Minimizing Equation (3), we obtain the equilibrium length

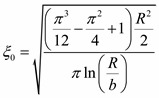
(4)

### 3.2. Electro- and Magneto-Optical Characteristics

The threshold character of the field dependences of the intensity of light passed through the samples (Figure 2, Figure 3, Figure 4, Figure 5 and Figure 6) and the absence of texture variations up to saturation voltages confirm our assumption that the field-assisted director reorientation in the nematic layer occurs without changing the structure of the LC surface domain layer [15]. It should be noted that determination of the Freedericksz threshold field using a classical technique [16] yields nonphysical results. In fact, under this assumption, at the constant of splay distortion *K*_11_ = 6.4 × 10^−7^ dyne and the dielectric anisotropy Δε = 13.3 [16], we obtain the calculated value *U*_1_' = π (4·π·*K*_11_/Δε)^1/2^ = 0.73 V for infinitely strong anchoring of the LC with surface. The experimental value of the threshold voltage is *U*_1_ = 1.2 V. However, *U*_1_ cannot be greater than *U*_1_' [17]. This is probably due to strong nonuniformity of the electric field in the LC with the strained transition layer with distance ξ_0_. In this situation, it is necessary to take into account additional contributions of the surface layer capacitance in strong electric field *E* [18] and the anisotropy of electric conductivity [19]. Since these contributions are very complex to be taken into account, we conducted additional experiments in a magnetic field that do not require field nonuniformity corrections. Thus, we established the correspondence between fields *E* and *H* (Figure 5 and Figure 6). It can be seen that the curves *I* (*U*) and *I* (*H*) nearly coincide at a normalization constant of 1.1. Therefore, the aligning effects of the electric and magnetic fields can be considered equivalent, so reduced field *H* can be used instead of field *E*. In this case, the experimental threshold magnetic field is *H*_1_ = 1.1·*U*_1_ = 1.3 kOe, if the bulk LC layer is assumed to be homogeneous. Under this assumption, at the magnetic susceptibility anisotropy constant Δχ = 1.18 × 10^−7^ [20], we obtain the calculated value *H*_1_' = π/*d* (*K*_11_/Δχ)^1/2^ = 2.4 kOe. The significant difference between *H*_1_' and *H*_1_ indicates that nematic anchoring with the PC surface is weak if polar energy *W*_θ_ used in the estimation is determined using a standard technique [21]. However, the adsorption of nematic molecules on the surface should cause a strong anchoring.

In addition, as shown in reference [10], the preferential adsorption of LC molecules on etched glass surfaces leads to the inability to use standard methods for the elastic interpretation of orientation effects. Evidently, the effects of adsorption reduce the voltage amplitude of the electric field with respect to the amplitude of the magnetic field in Figure 5 and Figure 6 in the oscillation regions of rapidly changing the orientation of the LC molecules. It is very likely that electrochemical reactions (such as an electron-transfer reaction between PC and 5CB molecules) are in the investigated structures. However, at higher voltage, no deviations of the saturation curves from the exponential *I* (*U*) dependency were observed. 

**Figure 8:**
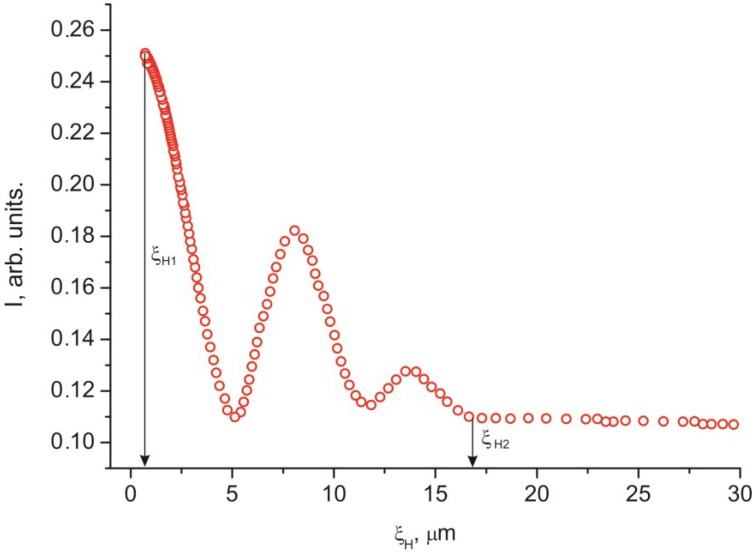
Intensity *I* of the laser radiation passed through the 30 µm thick planar 5CB layer with domains formed on the PC surface *versus* magnetic coherence length ξ_H,_ obtained from Figure 3. Lengths ξ_H1_ and ξ_H2_ correspond to voltages *U*_1_ and *U*_2_ related to the threshold field and limit optical resolution field.

The discrepancy between the experimentally determined threshold fields Fredericks *U*_1_ and *H*_1_ with the calculated values *U*_1_' and *H*_1_' confirms the unsuitability of the elastic model in the case of interaction of nematic with the PC surface. In this case we use the magnetic coherence length ξ_H_ [1] from the experimental dependences *I* (*H*). We will determine ξ_H_ by the expression ξ_H_ = 1/*H* (*K*/Δχ)^1/2^, where *K* = (*K*_11_ + *K*_22_ + *K*_33_)/3. Using the constants of torsion *K*_22_ = 3 × 10^−7^ dyne [20] and bend *K*_33_ = 8.6 × 10^−7^ dyne distortions [19], the electric field from Figure 3, and the correlations between the electric and magnetic fields in Figure 5 or Figure 6, we built the dependence *I* (ξ_H_) (Figure 8). The value of ξ_H_ that correspond to threshold voltage *U*_1_ = 1.2 V is ξ_H1_ ≈ 17 µm. The value ξ_H2_ ≈ 0.3 µm that correspond to voltage *U*_2_ is approximately equal to the He–Ne laser half-wavelength, *i.e.*, the optical resolution limit. In this sense, *U*_2_ is not a saturation voltage, which could be found by extrapolation of the smoothly varying initial portion of the curve *I* (ξ_H_) in the figure to the single LC layer.

**Figure 9:**
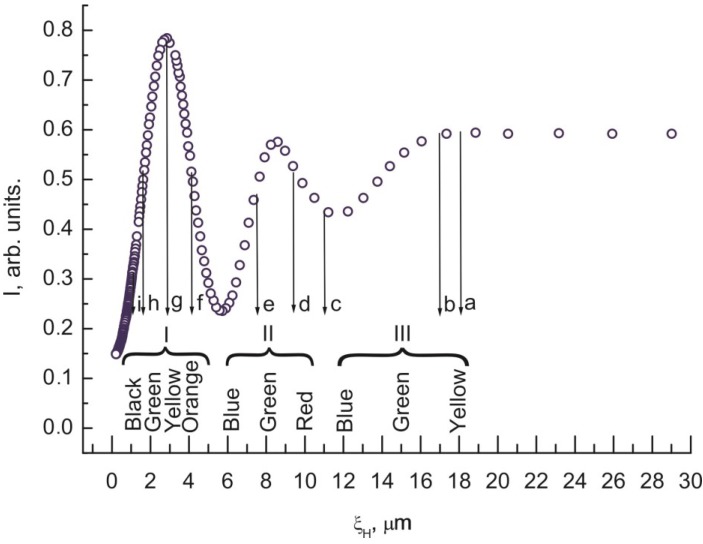
Dependence of intensity *I* of white light passed through the 30 µm thick planar 5CB layer with domains formed on the PC surface *versus* magnetic coherence length ξ_H_ obtained using Figure 2. Indices a–i correspond to Figure 1a,i. Designations black–yellow in the ξ_H_ scale correspond to the color sequence in the spectra of orders I–III obtained in an external magnetic field in the planar LC layer in the cell with glass surfaces.

Each of the dependences *I* (*U*) and *I* (*H*) in Figure 2, Figure 3, Figure 4, Figure 5 and Figure 6 contains a periodically varying portion with the light intensity oscillations. As can be seen in Figure 2, the oscillations occur despite the fact that the light penetrating through the LC layer is not polarized and comprises a set of electromagnetic waves of different wavelengths. Each curve contains two pronounced interference maxima and minima. Figure 9 presents the dependence *I* (ξ_H_) built using Figure 2. In the ξ_H_ axis, we added the black–yellow color scale obtained in the cell with the thickness δ = 30 µm with two plane-parallel glass surfaces rubbed to form uniform planar alignment of the nematic under the action of a magnetic field perpendicular to the surfaces. Designations a–i of the texture colors in Figure 1 correspond to the color sequence in the spectra of orders I–III of the homogeneous LC layer. This indicates the unambiguous correlation between the intensity of light passed through the LC layer and the magnetic coherence length. Therefore, we may state that in the bulk of the LC in an external field, the nematic direction changes its planar orientation for a uniform homeotropic one with a gradual decrease in the surface layer thickness.

The light intensity oscillations are caused by interference of light beams during their passage through the light-scattering surface layer. This is indicated by the coinciding optical transmission ranges in Figure 3 and Figure 4 in the cells with planar and homeotropic alignment in the bulk of the LC layer. The nature of probe radiation intensity oscillations is different from that of the phase modulation of polarized light caused by birefringence in the nematic layer, since the number of maxima and minima *m* in the *I* (*H*) curve obtained in the white light (Figure 2) equal *m* for nonpolarized light (Figure 5) and simply doubles in the experiment with the use of crossed polarizes (Figure 6). The oscillations apparently originate from the azimuth redistribution of the nematic director field from uniform alignment in the bulk to the radial configuration on the PC surface. The mechanism of light intensity oscillations will be investigated in the next study.

Thus, our experiments make us draw the following conclusions:

(i) The nematic orientation in the LC layer on the PC surface starts changing at a certain threshold electric or magnetic field.
(ii) In an external field, the nematic director in the bulk of the LC changes the uniform planar orientation for uniform homeotropic one as the surface layer thickness gradually decreases.
(iii) The orientation variations in an external field are accompanied by the light phase modulation, which can be caused by azimuth redistribution of the nematic director field from the uniform orientation in the bulk to the radial configuration on the PC surface.

**Figure 10:**
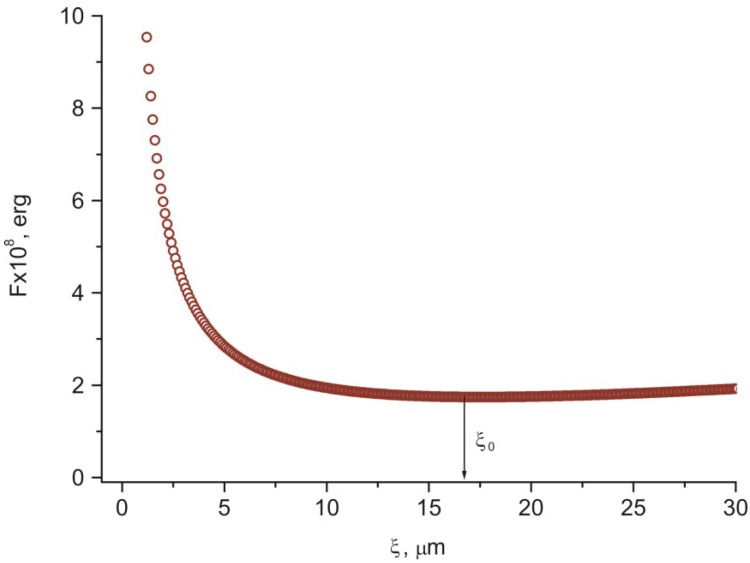
Dependence of free energy *F* on distance ξ from the PC surface. The arrow indicates equilibrium distance ξ_0_ that corresponds to the free energy minimum at the transition of the LC director field distribution from the radial configuration to uniform alignment in the bulk.

Figure 10 shows the dependence of free energy *F* on distance ξ from the PC surface, which was built using expression (3) and the experimental data *R* ≈ 85 µm and *b* ≈ 2 µm [15]. The equilibrium distance ξ_0_ ≈17 µm exactly determined using expression (4) coincides with the coherence length ξ_H1_ ≈ 17 µm in Figure 7 and Figure 8. One may expect that at ξ_H_ > ξ the director reorientation in the bulk of the LC layer will start at a gradual transition to the homeotropic orientation at ξ > ξ_H_.

## 4. Experimental Section

For the electro- and magneto-optical investigations, three types of LC cells were formed. In all the cells, the bottom substrate was a glass plate coated with a conducting indium tin oxide layer on top of which the 2% dichloromethane solution of the PC was centrifuged for a few tens of seconds to form the polymer film. The film placed two Teflon spacers and the top glass plate with the ITO coating. The first-type cell plate was chemically cleaned. The second-type cell top plate was coated with the 1% solution of lecithin to obtain homeotropic alignment. The third-type cell had symmetrical substrates coated with the PC film. In all the cells, the LC layer thickness was δ = 30 µm. We investigated the nematic LC 5CB. For spectral analysis, we additionally formed a cell with two 30 µm thick plane-parallel plates rubbed to establish planar nematic alignment. The cells were filled with the LC through a capillary spacing between the top and bottom plates in the nematic phase.

Domain structure formation in the investigated cells was observed on a BX51 polarizing microscope and a CAM V1200C/1.4M camera (Olympus Corporation, Shinjuku and West Medica and West Techno, Tokyo, Japan). The domain network formed in the cells for few minutes [15]. Observations in the polarizing microscope showed that in the first-type cells there are areas of uniform planar alignment. In these cells, the sample part with the planar radial LC structure was used. This structure was investigated by placing a diaphragm with a diameter of no more than the chosen part size on the probe beam path. In the second-type cells, homeotropic alignment was transferred from the top plate through the bulk LC layer and visualized the texture with radial domains near the bottom substrate surface. In these samples, we investigated the homeotropic radial structure [15]. In the third-type cells, planar nematic alignment was established using the memory effect observed during the formation of the LC structure in a magnetic field [21].

**Figure 11:**
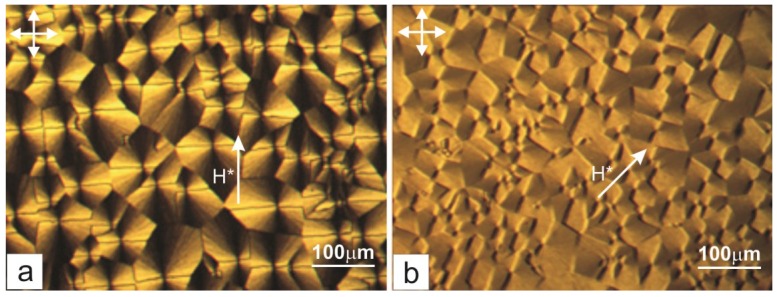
Microphotographs of the 5CB structure on the PC substrate parallel to which magnetic field H* was applied during the domain growth. (**a**) Direction of field H* coincides with the direction of one of the microscope polarizers (the polarizer axes are indicated by arrows); and (**b**) the direction of field H* makes an angle of 45° with the polarizer axis.

The effect consists of the following. In magnetic field H* applied to the sandwich parallel to the substrate with the PC film during the domain growth, uniform planar alignment occurred in the bulk of the nematic and remained after switching off the field (Figure 10). The orientation appeared stable against external electric and magnetic fields applied afterwards. The texture was pronounced when the direction of aligning field H* coincided with the direction of one of the microscope polarizers (Figure 11a) and brightened upon rotation of the cell by an angle of 45° (Figure 11b). This indicates the occurrence of the uniformly aligned layer in the bulk of the cell with the director parallel to field H*. The same effect was observed in the LC cells with symmetrical PC-coated plates.

In the electro- and magneto-optical investigations, the cells were formed at the temperature *T* = 24 °C. Schematics of the experiments are presented in the inserts in Figure 2, Figure 3, Figure 4, Figure 5 and Figure 6. In the experiments, radiation of an R-32734 He–Ne laser (Spectra-Physics, Santa Clara, CA, USA) L with the wavelength λ = 633 nm reflected from mirror M, passed through the sample and diaphragms D, and arrived to photodiode PD. If necessary, polarizers P were placed in the beam path at an angle of 45° to the nematic director. In the electrooptical experiments, voltage U with a generator Agilent 33250A (Agilent Technologies, Santa Clara, CA, USA) frequency of 1 kHz was applied to ITO electrodes. In the magnetooptical experiments, the samples were placed between the electromagnet poles.

## 5. Conclusions

The orientational transitions of the 5CB nematic in domain structures grown on the polycarbonate surface were investigated. In addition, the LC layers with the structures formed in a magnetic field applied parallel to the PC surface during the domain growth were studied. Electric- and magnetic-field dependences of the intensity of white and monochromatic light were established. The dependences of the light intensity on the magnetic coherence length were calculated. It was demonstrated that the field-assisted nematic alignment in the bulk of the LC layer on the PC surface start changing from the uniform planar orientation to the uniform homeotropic one at a certain threshold field value with a gradual decrease in the surface layer thickness. It was found that the field-assisted orientational variations are accompanied by the light intensity oscillations that can be attributed to the azimuth redistribution of the nematic director field from uniform alignment in the bulk to the radial configuration on the PC surface. The model was proposed that includes the equilibrium length extending from the surface to the bulk of the nematic layer, on which the radial structure continuously transforms to the uniform structure. The magnetic coherence length was compared with the equilibrium length.
